# Novel Ligands Targeting α_4_β_1_ Integrin: Therapeutic Applications and Perspectives

**DOI:** 10.3389/fchem.2019.00489

**Published:** 2019-07-09

**Authors:** Monica Baiula, Santi Spampinato, Luca Gentilucci, Alessandra Tolomelli

**Affiliations:** ^1^Department of Pharmacy and Biotechnology, University of Bologna, Bologna, Italy; ^2^Department of Chemistry “G. Ciamician,” University of Bologna, Bologna, Italy

**Keywords:** α_4_β_1_ integrin, agonist, antagonist, small molecules, inflammatory disorders

## Abstract

Among the other members of the adhesion molecules' family, α_4_β_1_ integrin, a heterodimeric receptor, plays a crucial role in inflammatory diseases, cancer development, metastasis and stem cell mobilization or retention. In many cases, its function in pathogenesis is not yet completely understood and investigations on ligand binding and related stabilization of active/inactive conformations still represent an important goal. For this reason, starting from the highlight of α_4_β_1_ functions in human pathologies, we report an overview of synthetic α_4_β_1_ integrin ligands under development as potential therapeutic agents. The small molecule library that we have selected represents a collection of lead compounds. These molecules are the object of future refinement in academic and industrial research, in order to achieve a fine tuning of α_4_β_1_ integrin regulation for the development of novel agents against pathologies still eluding an effective solution.

## Introduction

Integrins represent one of the most important families of cell adhesion receptors that mediate cell-cell and cell-extracellular matrix interactions. Integrins are heterodimeric transmembrane proteins composed by stable non-covalent association between α and β subunit. In mammals, 24 possible heterodimers have been identified, deriving from differential combination of 18 α subunits and 8 β subunits (Humphries et al., 2006).

**Figure 1:**
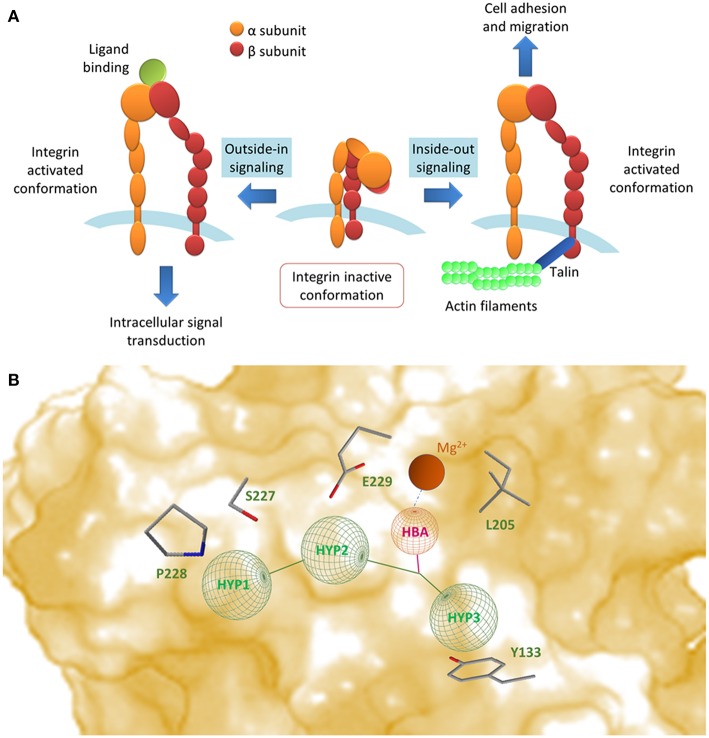
**(A)** Integrins propagate signals bidirectionally across cell membrane: conformational changes in their extracellular domains can occur as a consequence of signaling events happening inside the cells (inside-out signaling); these events lead to an increase in affinity (integrin activation) and therefore lead to ligand binding and cell adhesion. On the contrary, outside-in signaling represents the process in which ligand binding and ligand-induced integrin clustering lead to integrin-mediated intracellular signal transduction (Abram and Lowell, 2009; Ley et al., 2016). **(B)** Schematic representation of small molecule ligand binding mode to α_4_β_1_ integrin, obtained by combining the models reported in the literature. Side chains of selected residues in the β_1_ unit have been indicated. HBA, hydrogen bond acceptor; HYP1, valine mimetic hydrophobic pocket; HYP2-3, leucine mimetic hydrophobic pockets.

Integrins propagate signals bidirectionally across cell membranes (Abram and Lowell, 2009; Ley et al., 2016) (Figure 1A) and can be classified on the basis of the combination of α and β subunit (Tolomelli et al., 2017). The α_4_ subunit can couple with either β_7_ or β_1_ subunits. α_4_β_1_ integrin (also known as very late antigen-4, VLA-4) is expressed on leukocytes (lymphocytes, eosinophils, monocytes, macrophages, natural killer cells, basophils, and mast cells) and mediates homing, trafficking, differentiation, activation, and survival of α_4_β_1_ expressing cells (Hemler et al., 1987; Chan et al., 2001; Baiula et al., 2011; Mitroulis et al., 2015). VCAM-1 (vascular cell adhesion molecule-1), MAdCAM-1 (mucosal vascular addressin cell adhesion molecule-1), fibronectin and JAM-B (junctional adhesion molecule-B) are physiological ligands for α_4_β_1_ integrin (Imhof and Aurrand-Lions, 2004).

Targeting integrins has already proven to be a successful therapeutic strategy, with several agents approved for clinical practice. In this review, starting from the highlight of α_4_β_1_ functions in human pathology, we will give an overview of α_4_β_1_ integrin ligands under development as therapeutic agents.

## α_4_β_1_ Integrin as Therapeutic Target in Inflammatory Disorders

The α_4_β_1_ integrin plays a crucial role in inflammation. Extravasation is a multistep process consisting of leukocytes recruitment to inflamed tissue. The α_4_β_1_ integrin is mainly involved in the phases of leukocytes tethering and rolling on activated endothelial cells and in arrest and adhesion strengthening through the interaction with adhesion molecules such as VCAM-1 (Herter and Zarbock, 2019). Moreover, α_4_β_1_ integrin can bind JAM-B, expressed on endothelial cells, to enable transendothelial migration (Imhof and Aurrand-Lions, 2004). Therefore, targeting α_4_β_1_ integrin could be valuable for the treatment of inflammatory disorder, since α_4_β_1_ represents an absolute requirement for extravasation.

Of interest is the role of α_4_β_1_ in leukocyte homing to the CNS: this integrin is required for T cell migration across the blood brain barrier to the brain, and blocking α_4_β_1_ resulted in the inhibition of experimental autoimmune encephalitis (Kanwar et al., 2000).

In the following sections we will present a brief overview of the pathogenesis of the main inflammatory disorders involving α_4_β_1_ integrin.

### Multiple Sclerosis

Multiple sclerosis (MS) is a chronic inflammatory, autoimmune, demyelinating, and neurodegenerative disease of CNS. The pathogenesis of MS is very complex and not fully disclosed. Activated T lymphocytes are recruited from the blood into the CNS, through the interaction between α_4_β_1_ and VCAM-1, and release pro-inflammatory cytokines causing an inflammatory reaction that leads to neurodegeneration (Dargahi et al., 2017). Blocking α_4_ integrin results in inhibition of trafficking of T cells from the blood to CNS: natalizumab is a humanized monoclonal antibody (mAb) that binds to α_4_ subunit and thus blocks both α_4_β_1_ and α_4_β_7_; it has been approved for the treatment of highly active relapsing and remitting MS (Clerico et al., 2017) and Crohn's disease (see below). However, progressive multifocal encephalopathy (PML) occurred as a fatal adverse effect of natalizumab (Shirani and Stüve, 2017).

### Inflammatory Bowel Diseases

Inflammatory bowel diseases (IBD), including ulcerative colitis (UC), and Crohn's disease (CD), are chronic relapsing inflammatory disorders of the gut (Zundler et al., 2017a). IBD pathogenesis is not completely understood, and comprises several factors: among them, infiltration of immune cells in the gut plays a pivotal role. T lymphocytes homing to the gut is mainly mediated by α_4_β_7_-MAdCAM-1 interaction. Consequently, vedolizumab, a humanized mAb anti-α_4_β_7_, has been developed and approved for the treatment of both UC and CD (Feagan et al., 2013; Sandborn et al., 2013). In addition, α_4_β_1_ integrin contributes to the infiltration of T cells to the inflamed intestinal tissue (Zundler et al., 2017b). Natalizumab, targeting α_4_ integrin subunit, has been approved for the treatment of MS and for CD, although due to fatal adverse reactions, its use is very limited for CD (Li et al., 2018; Nelson et al., 2018). Recently it has been observed that vedolizumab did not cause a decrement in homing of T cells into the gut, which instead was achieved by blocking α_4_β_1_. These data suggest that α_4_β_1_-dependent homing can represent a compensatory mechanism to evade α_4_β_7_ blockade. It is not still known if this mechanism is clinically relevant for CD (Zundler et al., 2017b).

### Allergic Conjunctivitis

Allergic conjunctivitis is the most common form of ocular allergy (Baiula et al., 2011; Baiula and Spampinato, 2014). This disease is mainly characterized by an inflammatory response of the conjunctival mucosa that leads, through the interaction of integrins with adhesion molecules, to a long-term infiltration of neutrophils, eosinophils and T lymphocytes. Integrin α_4_β_1_ is strongly involved in the recruitment of circulating cells at the inflamed conjunctiva, contributing to both rolling and firm adhesion (Bacon et al., 1998). The reduction of α_4_β_1_ expression at conjunctival level, is part of the mechanism of action of the antihistamine levocabastine (Qasem et al., 2008).

### Dry Eye Disease

Dry eye disease (DED) is a common cause of ocular discomfort and visual disturbance (Miljanović et al., 2007). DED is associated with ocular surface inflammation characterized by infiltration of T cells and overexpression of inflammatory mediators although the pathogenesis is not fully understood. α_4_β_1_ integrin blockade, using small molecule α_4_β_1_ antagonists, strongly reduced T cell infiltration into the ocular surface and ameliorated ocular signs in *in vivo* models of DED (Ecoiffier et al., 2008; Krauss et al., 2015).

### Asthma

Asthma is a chronic inflammatory disease of the lower respiratory tract (Mims, 2015). α_4_β_1_ integrin, expressed on inflammatory cells, participates in the pathogenesis of asthma (Ohashi et al., 1992) and sarcoidosis, a disorder characterized by lymphocyte accumulation in the lung (Berlin et al., 1998). Several α_4_β_1_ antagonists have been developed but they lack efficacy in clinical trials (Teoh et al., 2015).

### Stem Cell Mobilization or Retention

Hematopoietic stem cell (HSC) express several integrins, including α_4_β_1_ which is involved in the regulation of HSC homing and retention within the bone marrow niche (Grassinger et al., 2009). Novel agents able to mobilize HSC and progenitor cells are actively searched and clinically important to obtain cells from healthy donors for transplantation. The blockade of α_4_β_1_/α_9_β_1_ with a dual antagonist induced a rapid and transient mobilization of HSC (Cao et al., 2016). Moreover, bortezomib, a proteasome inhibitor that blocks the expression of VCAM-1, had a mobilizing effect by the modulation of α_4_β_1_/VCAM-1 axis (Ghobadi et al., 2014). This strategy based on the blockade of α_4_β_1_ integrin has shown great promise also for HSC transplantation *in utero*.

On the contrary, the activation of α_4_β_1_ may be a promising strategy to improve cell retention and engraftment in stem cell-based therapies (Vanderslice et al., 2013). The homing of endothelial progenitor cells to sites of ischemia, regulated by α_4_β_1_ integrin, has been shown to promote neovascularization in ischemic tissue (Duan et al., 2006).

### Cancer and Metastasis

Several types of tumor cells express α_4_β_1_ integrin and the interaction with its ligand VCAM-1 increases transendothelial migration and contributes to metastasis to distant organs (Schlesinger and Bendas, 2015). Moreover, an aberrant expression of VCAM-1 has been observed in tumor cells. In breast cancer cells, VCAM-1 seems to confer an increased ability to metastasize to the bones and the lungs (Vanharanta and Massagu, 2013). In addition, α_4_β_1_ plays an important role in tumor angiogenesis, as do other integrins (Gentilucci et al., 2010), and in the development of drug resistance (Schlesinger and Bendas, 2015).

Recent evidences hypothesize an apparent tumor-protective role of α_4_β_1_ integrin in a mouse model of colon adenocarcinoma: when α_4_β_1_ was depleted, an accelerated tumor growth was observed (Oh et al., 2018). Considering these preliminary results, authors suggest manipulation of α_4_β_1_ levels could be achieved using small molecule agonists.

## Small Molecules Targeting α_4_β_1_ Integrin

Small molecules selectively binding to α_4_β_1_ integrin have been designed on the basis of the minimal recognition sequences with the extracellular matrix proteins. In particular, the tripeptide LDV (Leu-Asp-Val) that has been recognized as the binding sequence found in the alternatively spliced connecting segment (CS1) region of fibronectin (Komoriya et al., 1991), is homologous and quite isosteric to the fragment IDS (Ile-Asp-Ser), present in the binding site of VCAM-1 to α_4_β_1_.

Due to the lack of crystal structures of ligand-receptor complexes (Jones et al., 1995), suggestions on the required three-dimensional features that may ensure optimal affinity have been deduced only by combining homology models deduced by the template of β_2_ integrins (CD11A/CD11B) (You et al., 2002), QSAR studies on small library of LDV mimicking ligands (Singh et al., 2002; Hutt et al., 2011; Thangapandian et al., 2011; Amin et al., 2018), molecular dynamics and ligand-receptor docking studies (Silva et al., 2010). In general, effective ligands should possess a hydrogen bond acceptor, typically a carboxylate moiety, to coordinate the metal cation in the β unit and lipophilic groups that find accommodation into the pockets usually occupied by valine and leucine side chains (Figure 1B). Three complete and detailed overviews on α_4_β_1_ synthetic ligands have already been reported (Jackson, 2002; Tilley, 2002; Huryn et al., 2004), but a collection of the more recent results in the design and synthesis of these bioactive compounds is lacking. We report herein a selection of the most recent examples of bioactive small molecule ligands to α_4_β_1_ integrin. The structures of the cited compounds are reported in Table 1.

**Table 1:** A collection of lead compounds, ligands of α_4_β_1_ integrin.

**Entry**	**Structure**	**IC_**50**_/EC_**50**_ (nM)**	**Biological assay**	**References**
1	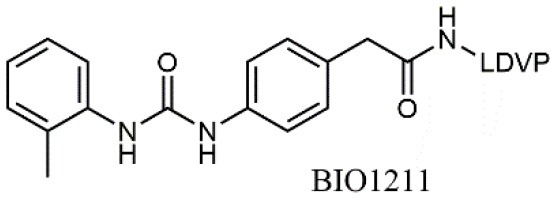	4.0	Jurkat cell Mn^2+^-induced adhesion to VCAM-Ig-AP	Lin et al., 1999
2	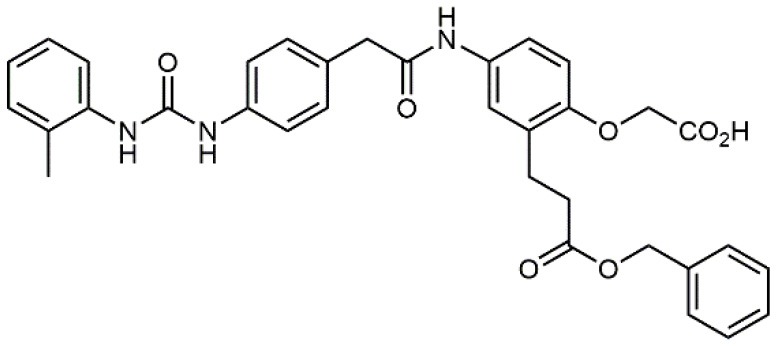	24,300 ± 4,500	CCRF-CEM cell adhesion to fibronectin	Gérard et al., 2012a
3	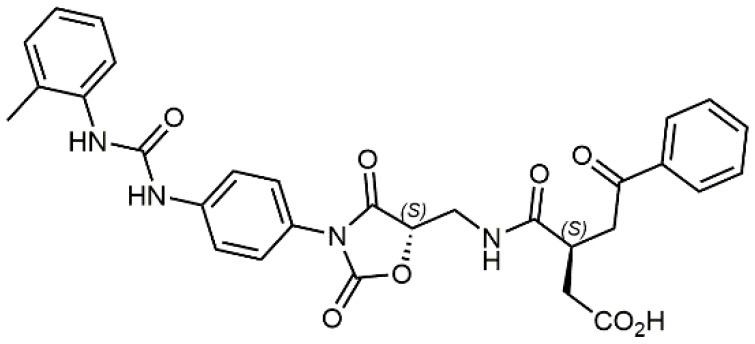	19 ± 20	Jurkat cell adhesion to VCAM-Ig-AP	De Marco et al., 2015
4	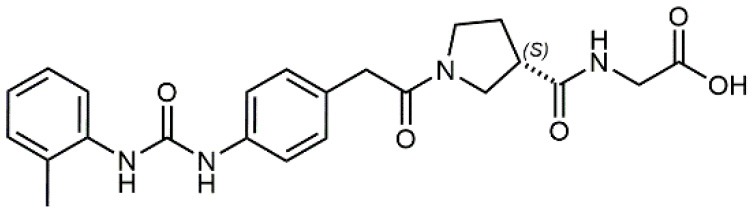	5.04 ± 0.51 *antagonist*	Jurkat cell adhesion to VCAM-1	Dattoli et al., 2018
5	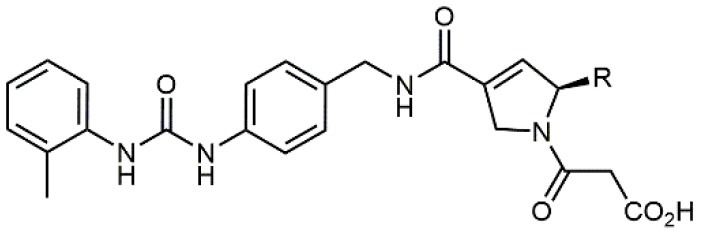	10 ± 3 *antagonist*	Jurkat cell adhesion to VCAM-1	Tolomelli et al., 2015
6	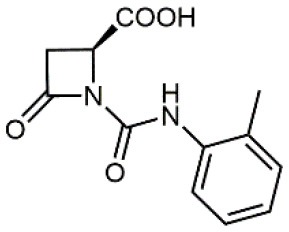	1.39 ± 0.04 *antagonist*	Jurkat cell adhesion to VCAM-1	Baiula et al., 2016
7	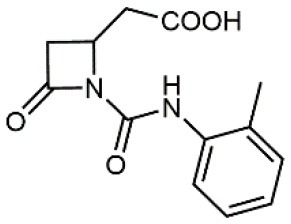	12.9 ± 0.6 *agonist*	Jurkat cell adhesion to VCAM-1	
8	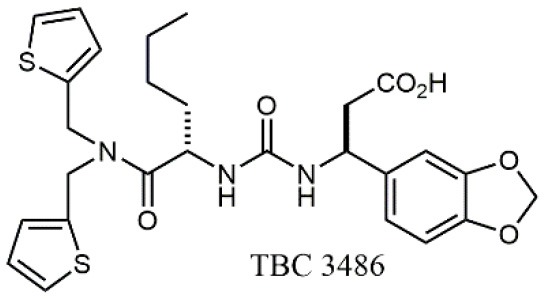	9.0 *antagonist*	K562-$\alpha_{4}\beta_{1}^{+}$ cell adhesion to VCAM-1-Ig	Vanderslice et al., 2010
9	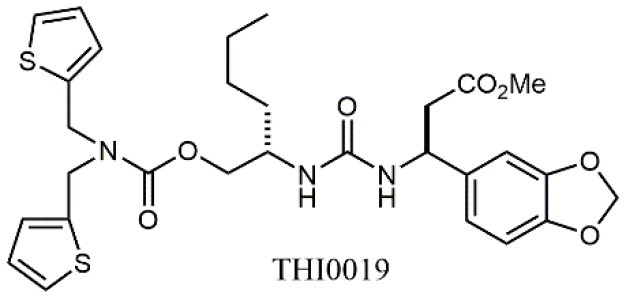	1,000 *agonist*	K562-$\alpha_{4}\beta_{1}^{+}$ cell adhesion to VCAM-1-Ig	Vanderslice et al., 2013
10	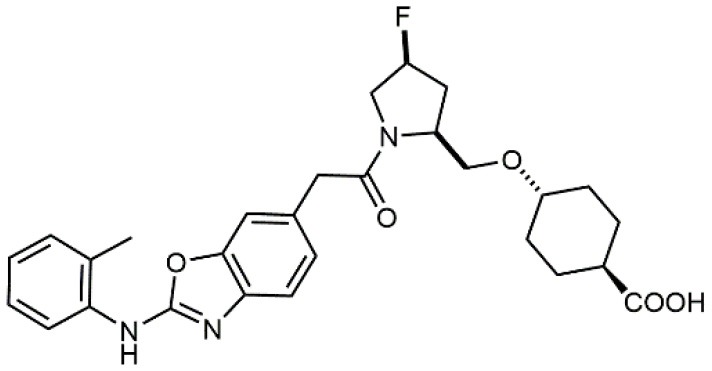	2.8	VLA-4/Eu-Human VCAM-1 binding assay	Muro et al., 2009
11	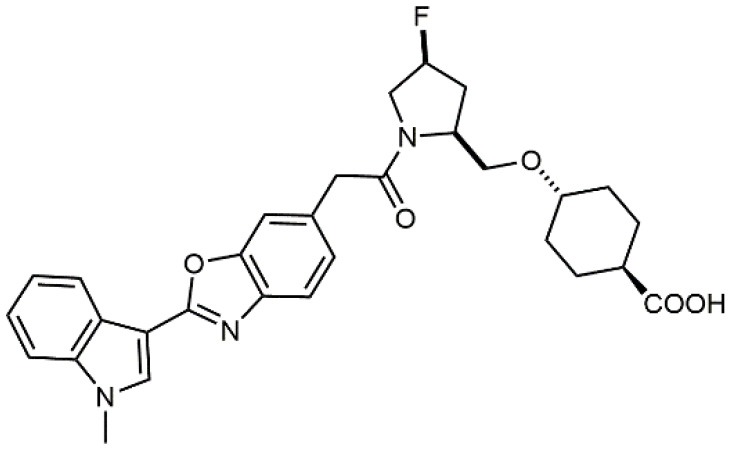	4.7	VLA-4/Eu-Human VCAM-1 binding assay	Setoguchi et al., 2012
12	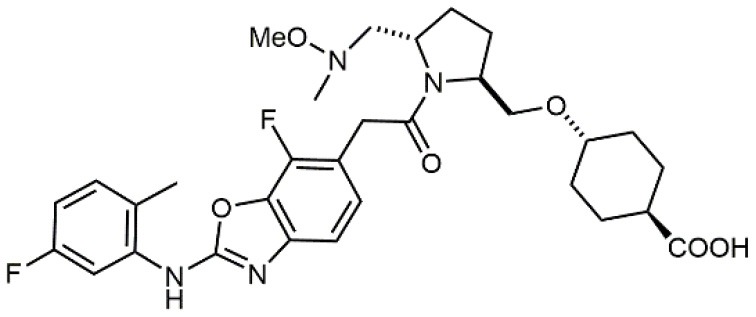	1.7	VLA-4/Eu-Human VCAM-1 binding assay	Setoguchi et al., 2013
13	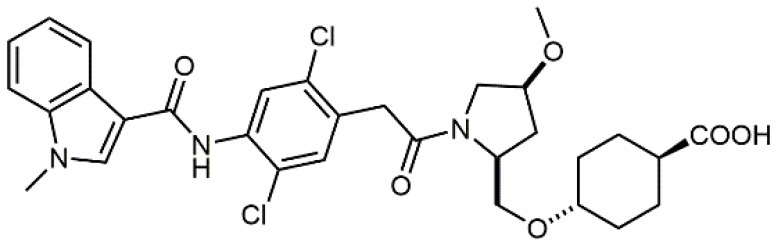	In clinical development		Kapp et al., 2013
14	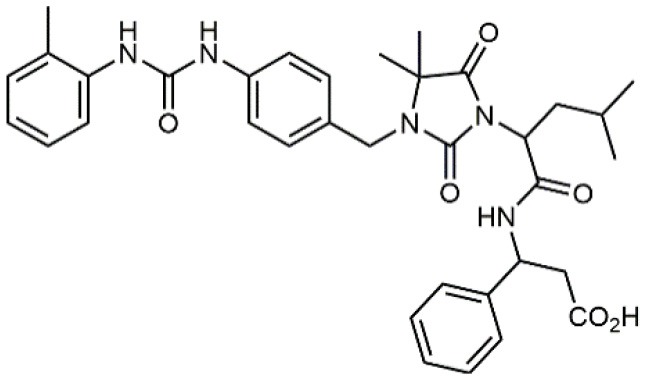	/		Crofts et al., 2004
15	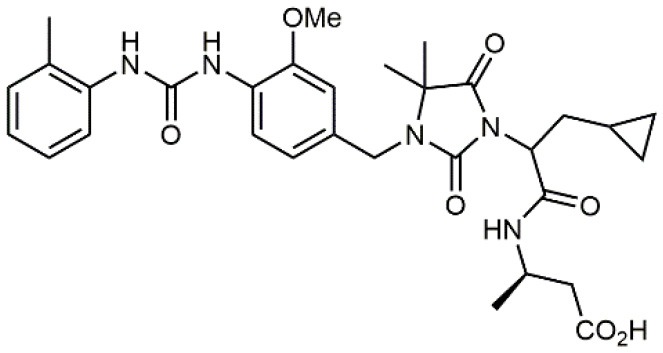	0.29	VCAM-1-IgG adhesion assay to U937 cells	Gläsner et al., 2005
16	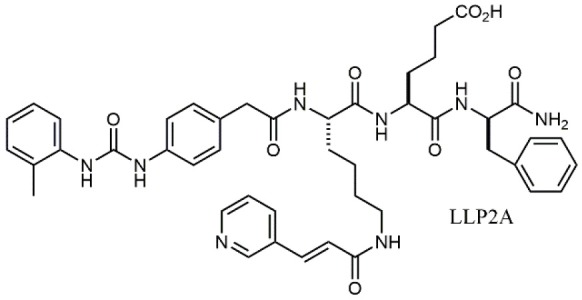	0.002	Jurkat cell adhesion to CS-1 peptide	Peng et al., 2006
17	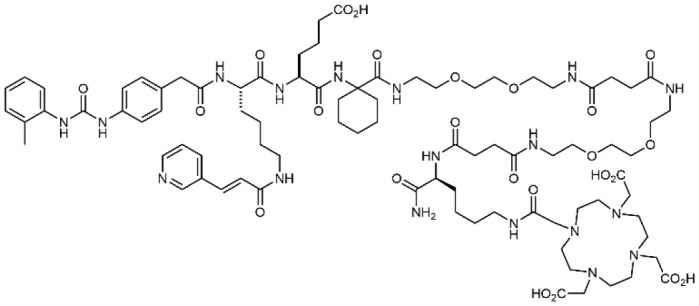	/		Denardo et al., 2009
18	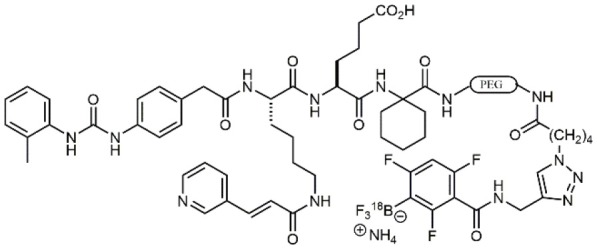	/		Walker et al., 2016
19	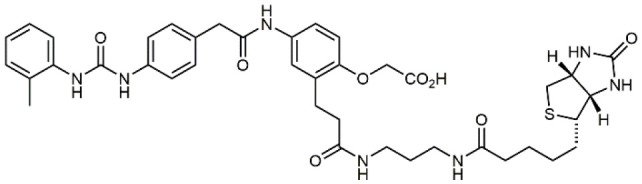	14,000	fCCRF-CEM leukemia cell adhesion to fibronectin	Gérard et al., 2012b
20	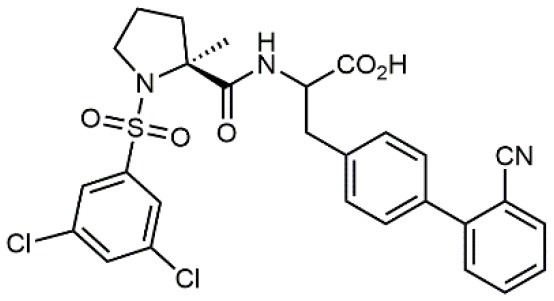	0.92	^125^I-VCAM-Ig to VLA-4 binding assay	Hagmann et al., 2001
21	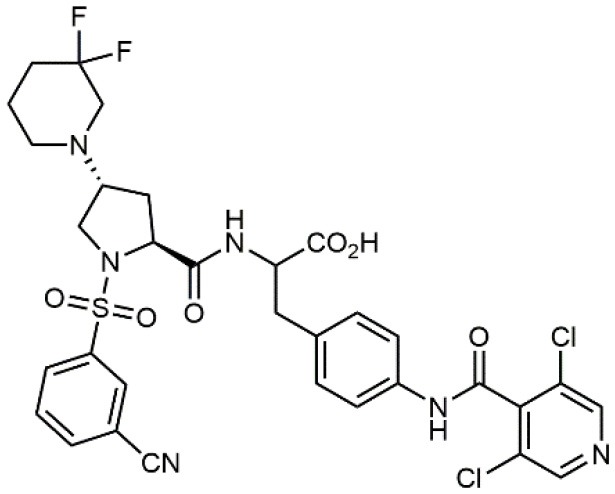	0.03 ± 0.01	^125^I-VCAM-1 binding assay to Jurkat cells	Venkatraman et al., 2009
22	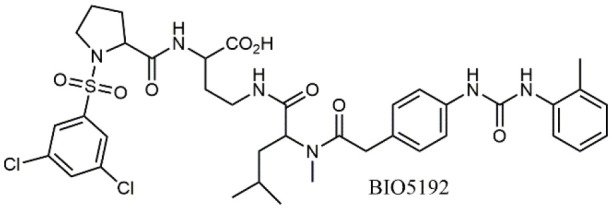	1.8	Jurkat T-cell leukemia cell adhesion to fibronectin	Ramirez et al., 2009
23	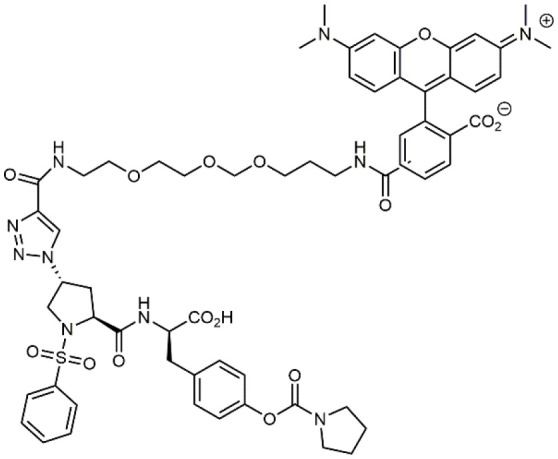	20.1	Saturation binding experiments LN18-$\alpha_{4}\beta_{1}^{+}$cells	Cao et al., 2014
24	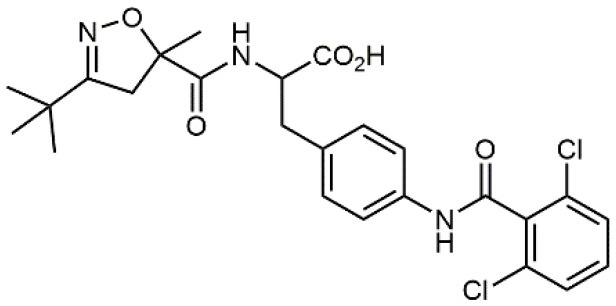	4 ± 2	U937 T cell adhesion to VCAM-1	Soni et al., 2013
25	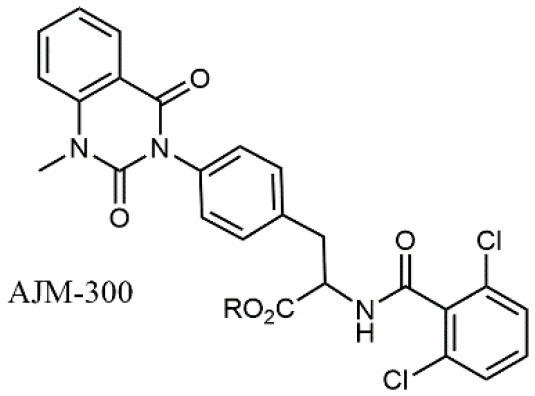	5.8 ± 1.6	Jurkat T-cell adhesion to hVCAM-1/Fc	Sugiura et al., 2013
26	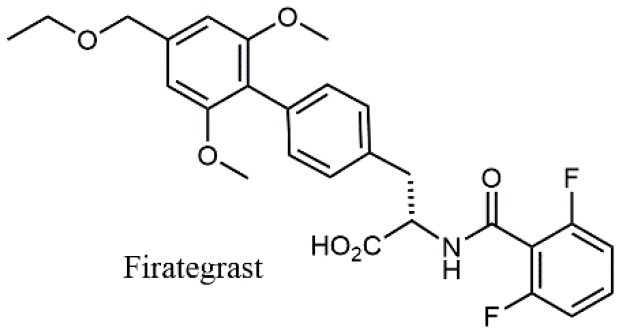	/		Kawaguchi et al., 2002; Kim et al., 2016
27	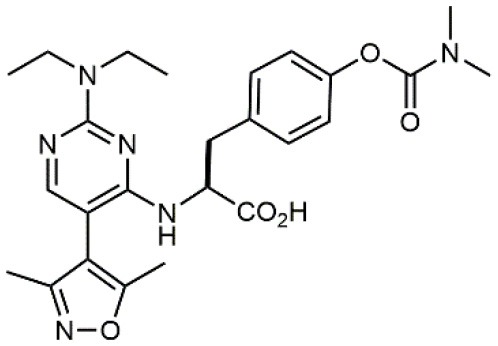	1.0	Jurkat cell to recombinant VCAM-1 FACS assay	Semko et al., 2011
28	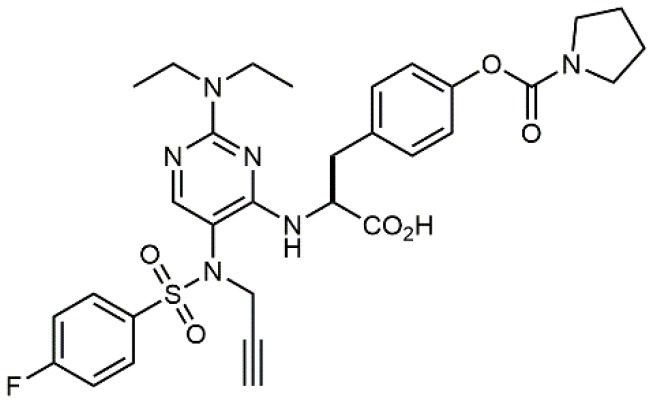	8.0	Jurkat cell adhesion to fibronectin	Xu et al., 2013a,b
29	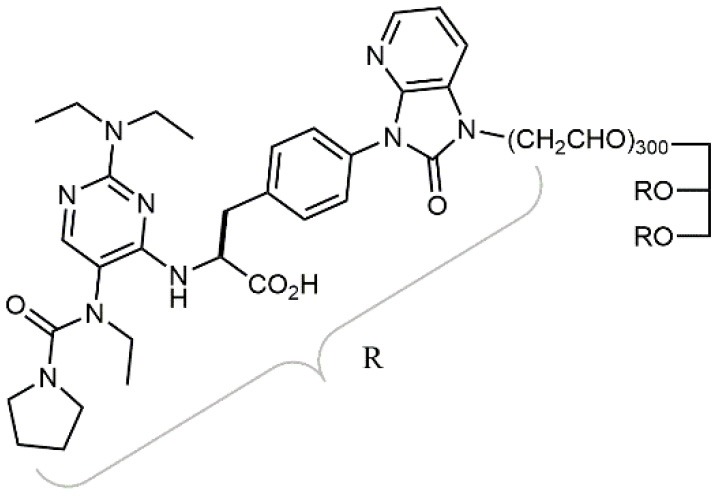	1.7	Jurkat cell adhesion to VCAM-1	Smith et al., 2013
30	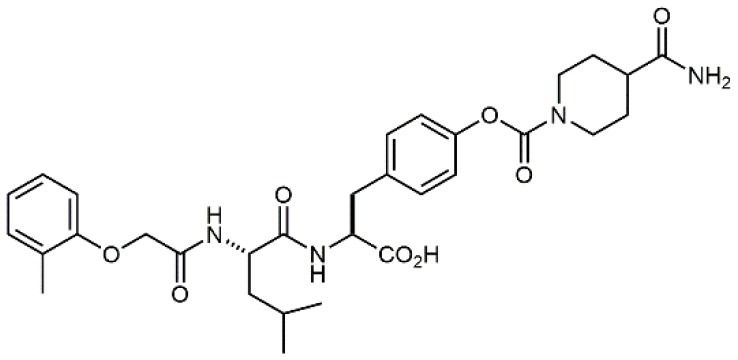	7.72	Jurkat J6 cell (human lymphoblast cell line) adhesion to VCAM-1	Krauss et al., 2015
31	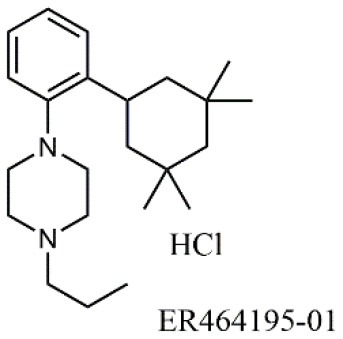	150	PMA-induced T cell adhesion to VCAM-1	Ohkuro et al., 2018
32	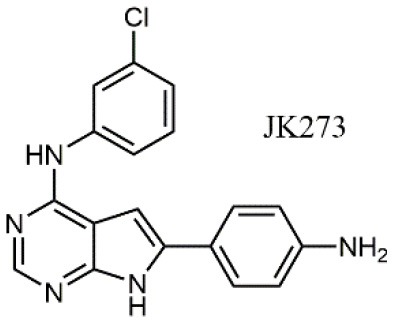	1,000–5,000	PMA-induced Jurkat/U937 cell adhesion to fibronectin	Lee et al., 2009
33	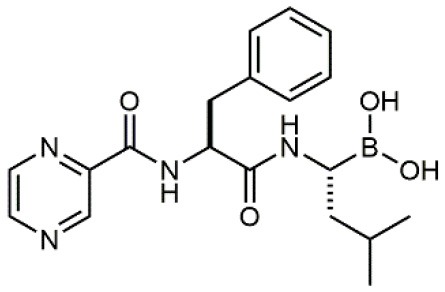	/		Noborio-Hatano et al., 2009; Ghobadi et al., 2014

The biological assays in which they have been tested in vitro and their potency/affinity (nM) are reported.

### N-benziloxycarbamido Phenylurea (PUPA) Containing Ligands

Starting from the LDV recognition sequence, Adams et al. (Lin et al., 1999) synthesized a small library of oligopeptides which had the terminal amino group capped with benziloxycarbamido phenylurea (PUPA). Introduction of this moiety allowed to identify compound BIO1211 (*N*-PUPA-LDVP), which is 10^6^ fold more potent than the corresponding peptide and possesses an enhanced resistance toward enzymatic hydrolysis. On this basis, *N*-PUPA containing linear peptidomimetics, respecting the fundamental requirements for affinity, showed micromolar inhibitory activity on fibronectin adhesion to human T lymphoblast like cells (Gérard et al., 2012a). Introduction of five membered rings as amide bond isosters and conformational restraints have been exploited by several groups. Recently, 5-aminomethyloxazolidine-2,4-dione (Amo) dipeptide scaffold, analog of the well-known Freidinger lactam, was successfully introduced as a central core into peptidomimetics, designed to maintain a 14-bond carboxylate-urea distance, displaying nanomolar IC_50_ in cell adhesion assays (De Marco et al., 2015). On the other hand, since proline has been often inserted into peptide sequences to induce specific conformations, (D)-configured β2-proline-containing ligand DS70 was synthesized and successfully tested in cell assays and in a guinea pig model of allergic conjunctivitis (Dattoli et al., 2018). A small library of compounds containing the rigid dehydro-β-proline ring also showed excellent affinity to α_4_β_1_ integrin (Tolomelli et al., 2015). For these compounds, a strong dependence on the stereochemistry of the heterocyclic central core was observed, thus suggesting a specific disposition of the lipophilic chain for the two enantiomers. Decorating the simpler four membered β-lactam scaffold also afforded effective ligands. In particular, agonists and antagonists to α_4_β_1_ were identified, by evaluating their ability to inhibit or activate cell adhesion. This behavior was ascribed to their ability to promote and stabilize active or inactive conformations of the receptor (Baiula et al., 2016). A previous study already suggested that small modifications in ligand structure could induce dramatic effect on their agonist/antagonist behavior. Compound TBC3486, a potent nanomolar antagonist selective for α_4_β_1_ integrin (Vanderslice et al., 2010), was converted into THI0019, a micromolar agonist, simply by introducing an oxymethylene bond and protecting the carboxylic terminal as methyl ester. Stabilization of an active conformation by the agonist, followed by its fast displacement was suggested to justify this result (Vanderslice et al., 2013).

Increased bioavailability was obtained by Daiichi Sankyo Ltd. researchers by linking fluoro-prolinol derivatives to halogen or alkyl substituted aromatic PUPA fragment (Muro et al., 2009). Benzoic or cyclohexanecarboxylic acids were introduced in this class of compounds as metal binding pharmacophores (Setoguchi et al., 2012). Modifications to the polar surface and the number of hydrogen bonds by changing PUPA with other lipophilic groups has also been explored (Setoguchi et al., 2013). Selected members of this library are currently in clinical development (Kapp et al., 2013). Another PUPA-containing ligand, HMR 1031, was enrolled by Aventis Pharmaceuticals to phase II clinical trials, but the suspect risk of teratogenicity decreased the interest on this compound (Crofts et al., 2004). Anyway, a similar molecule was lately reported to prevent development of arthritis in Lyme disease infection (Gläsner et al., 2005).

### N-PUPA Derivatives for Imaging Application

In the last few years, due to the drawbacks of some promising integrin targeting compounds *in vivo*, beside the therapeutic applications, the use of selective ligands as imaging agents has been widely explored. For instance, the peptidomimetic LLP2A, displaying extraordinarily high affinity (IC_50_ = 2 pM) to the α_4_β_1_ integrin receptor (Peng et al., 2006), was conjugated with NIR tags through a PEG linker and applied to the detection of MOLT-4 tumor xenografts. Similar derivatives were linked to radioactive metal chelators for *in vivo* imaging (Denardo et al., 2009; Gai et al., 2018). More recently, ^18^F-labeled LLP2A-trifluoroborate bioconjugates were successfully evaluated in α_4_β_1_ integrin-overexpressing tumor models (Walker et al., 2016). Bioconjugation of BIO1211 derivatives with biotin though linear spacer arms allowed the exploitation of the extraordinary affinity with streptavidin/avidin coated supports to detect and capture leukemia cells overexpressing α_4_β_1_ (Gérard et al., 2012b).

### Proline-Phenylalanine Dipeptide Deriving Ligands

A second important family of small molecule ligands possesses, as a common feature, the presence of an *N*-acylated para-substituted phenylalanine core. Among them, proline-phenylalanine derivatives showed excellent activity, lacking unfortunately of satisfactory bioavailability due to their peptidic nature. For this reason, much attention has been devoted to obtain a better pharmacokinetic profile and impart oral availability. Arylsolfonamide proline dipeptides (Hagmann et al., 2001), discovered from directed screening of a combinatorial library, showed picomolar α_4_β_1_ affinity, but unfortunately these compounds were very rapidly cleared from plasma. Thus, a novel series of potent prolyl dipeptide α_4_β_1_ antagonists containing fluorinated cyclic tertiary amines at the proline 4-position was developed. In general, the fluorinated compounds provided improved potency when compared with their des-fluoro analogs (Venkatraman et al., 2009). The highly potent BIO5192 (Ramirez et al., 2009) ligand shares some common features with the above reported molecules, as it is a chimera between arylsolfonamide proline dipeptides and PUPA-substituted compounds. This molecule displayed the ability to mobilize hematopoietic stem and progenitor cells (HSPC) but was not selected for clinical development. Conjugation of already reported *N*-phenylsulfonyl proline-based integrin antagonists (Pepinsky et al., 2002) to PEG-linker or fluorophore afforded novel compounds, exhibiting high nanomolar dual binding affinities to α_9_β_1_ and α_4_β_1_ integrins. Furthermore, these ligands are capable of binding haemopoietic progenitor cells and HSC within mice bone marrow *in vivo* (Cao et al., 2014).

By replacing the proline ring with a 3-alkyl-isoxazoline-5-carboxamide, ligands showing nanomolar activity and possessing stability in microsomes were obtained (Soni et al., 2013). Compound TR14035, first reported in 2002 by Tanabe, is a dichloro-substituted benzamides of biphenylalanine scaffold. This molecule displayed nanomolar IC_50_, but acted as a dual ligand, being active both on α_4_β_1_ and α_4_β_7_ integrins. Anyway, it represented the lead compound for the development of refined derivatives, such as AJM-300, developed by Ajinomoto, where the biphenyl chain was replaced by a phenyl-pirimidindione. This compound is currently in clinical phase III for ulcerative colitis (Sugiura et al., 2013). Firategrast (Kawaguchi et al., 2002), developed by Tanabe and GSK, belonging to the same class of compounds, reached phase II trials for MS and is currently studied as a facilitator in the “*in utero*” hematopoietic cell transplantation (IUHCT), a pioneering approach for critical fetal diseases treatment (Kim et al., 2016). By refining the structure of an already reported antagonist α_4_β_1_, the researchers of Elan Pharmaceuticals faced the limited bioavailability of their lead compound, by introducing *N*-arylated heterocycles to mimic the carboxamide (Semko et al., 2011). This last moiety was indeed considered partially responsible for the poor pharmacokinetic profile. As a result, they identified a novel compound displaying a greatly improved pharmacokinetic profile and robust efficacy in a sheep asthma model (Xu et al., 2013a). In further investigations, the same structure was properly modified achieving excellent bioavailability and, in some cases, dual affinity for α_4_β_1_ and α_4_β_7_ integrins (Xu et al., 2013b). Linking these molecules at each of the termini of a three-arm branched PEG provided potent *in vivo* α_4_ integrin inhibitors (Smith et al., 2013). In general, due to the high similarity of the α_4_β_1_ and α_4_β_7_ integrins receptors, several cases of dual ligands have been reported in the literature (Tilley et al., 2013). Finally, among the phenylalanine containing dipeptides, particular interest has been recently paid to GW559090, for its effects on corneal staining and ocular surface inflammation in murine model of DED (Krauss et al., 2015).

### Tellurium Compound

AS101 [ammonium trichloro(dioxoethylene-o,o′)tellurate], a small tellurium^IV^ compound, has been shown to inhibit α_4_β_1_ function by redox inactivation of adjacent thiols in the extracellular domain of α_4_β_1_ (Smith et al., 2002; Chigaev et al., 2004). Since this small molecule is not mimicking the recognition sequences in the extracellular matrix proteins, it has no structural similarity with all the other ligands. Through the regulation of integrin functions and immunomodulatory properties, AS101 significantly reduced clinical manifestations of IBD and other autoimmune and inflammatory diseases (Halpert et al., 2014).

### Ligands Indirectly Regulating α_4_β_1_ Integrin Activity

The activity of α_4_β_1_ integrin may also be controlled by acting on other biomolecules. For instance, orally active ER464195-01, an antagonist to calreticulin (CRT), has been reported as an inhibitor of VCAM-1 mediated cell adhesion (Ohkuro et al., 2018), having an IC_50_ in the μM range. The effect is indirect since CRT is a calcium binding chaperone involved in integrin α subunit activation. On the other hand, targeting γ-parvin, a component of focal adhesions involved in the downstream of α_4_ integrin, allowed for the identification of compound JK273 (Lee et al., 2009), as an alternative modulator of α_4_ integrin mediated leukocyte trafficking.

## Concluding Remarks

Involvement of α_4_β_1_ integrin in several diseases still waiting for an efficacious treatment and for a clear understanding of their pathogenesis, confirms the importance of this receptor as a therapeutic target. Already developed agents in late-phase clinical trial offer great expectations to patients, but the discovery of novel small molecule ligands, targeting not only binding but also selective conformation in active/inactive state, may offer great potential for novel treatments. Improvement of pharmacokinetic and pharmacodynamic properties of compounds that could allow managing of oral therapies in home environment is still an issue. Anyway, the use of α_4_β_1_ integrin ligands in bioconjugation with imaging agents is also a field of growing interest.

Finally, the recognized role of α_4_β_1_ integrin in the regulation of HSC homing, retention and engraftment suggests a paramount role of these receptor in the future development of post-transplantation treatments and prenatal therapy of fetus pathologies.

## Author Contributions

MB, SS, and AT equally contributed to the preparation of the manuscript and LG approved the final version.

### Conflict of Interest Statement

The authors declare that the research was conducted in the absence of any commercial or financial relationships that could be construed as a potential conflict of interest.

## Abbreviations

VLA-4: very late antigen-4
VCAM-1: vascular cell adhesion molecule-1
MAdCAM-1: mucosal vascular addressin cell adhesion molecule-1
JAM-B: junctional adhesion molecule-B
MS: multiple sclerosis
IBD: inflammatory bowel diseases
DED: dry eye disease
CNS: central nervous system
PML: progressive multifocal encephalopathy
UC: ulcerative colitis
CD: Crohn's disease
mAb: monoclonal antibody
ICAM-1: intercellular adhesion molecule-1
HSC: hematopoietic stem cell
LDV: Leu-Asp-Val
IDS: Ile-Asp-Ser
QSAR: quantitative structure-activity relationship
PUPA: benziloxycarbamido phenylurea
PEG: polyethylene glycol
HSPC: hematopoietic stem and progenitor cells
IUHCT: *in utero* hematopoietic cell transplantation
CRT: calreticulin.
